# Histomorphometric Evaluation of Superovulation Effect on Follicular Development after Autologous Ovarian Transplantation in Mice

**DOI:** 10.1155/2015/236436

**Published:** 2015-11-26

**Authors:** Amin Tamadon, Alireza Raayat Jahromi, Farhad Rahmanifar, Mohammad Ayaseh, Omid Koohi-Hosseinabadi, Reza Moghiminasr

**Affiliations:** ^1^Transgenic Technology Research Center, Shiraz University of Medical Sciences, Shiraz, Iran; ^2^Department of Clinical Sciences, School of Veterinary Medicine, Shiraz University, P.O. Box 1731-71345, Shiraz, Iran; ^3^Department of Basic Sciences, School of Veterinary Medicine, Shiraz University, P.O. Box 1731-71345, Shiraz, Iran; ^4^Laboratory Animal Center, Shiraz University of Medical Sciences, Shiraz, Iran; ^5^Department of Stem Cells and Developmental Biology, Cell Science Research Center, Royan Institute for Stem Cell Biology and Technology, ACECR, Tehran, Iran

## Abstract

The effect of superovulation by pregnant mare serum gonadotropin (PMSG) on autologous transplanted ovaries in the lumbar muscles of mice was histomorphometrically evaluated using the indices of number and volume of different kind of follicles and volume of corpora lutea, ovary, and stroma. Angiogenesis was observed after mouse ovarian transplantation on days 14 and 21 after ovarian grafting. After transplantation, the total number and volume of primary and secondary follicles reduced, while PMSG superovulation increased the total number and total volume of tertiary follicles and also the ovarian volume after transplantation. Transplantation increased the average size of primary, secondary, and tertiary follicles. Therefore, primary and secondary follicles can survive after autologous transplantation but their reservations diminished by increasing the time of transplantation. However, number of tertiary follicles and their response to superovulation increased over time after transplantation.

## 1. Introduction

Ovary transplantation is a method for preservation of endangered and valuable species [1]. On the other hand, ovarian transplantation has the potential application for maintaining the fertility after chemotherapy and radiotherapy in women [2]. As a result of the ovarian transplantation, the possible depletion of follicle reserve and limitation of fertility restoration exist [3]. The major concern in grafting is that the graft survival is completely dependent on the establishment of neovascularization [4]. A number of follicles may be lost because of hypoxia and ischemia. For evaluation of the effect of ischemia after ovarian transplantation, whole or piece of small ovaries of laboratory rodents can be used [5]. To prevent ischemia and increase the rate of angiogenesis, surgery must be rapid and the ovarian tissue should be placed in a highly vascular tissue [6]. It is shown that ischemia may cause disappearance of 50% or even greater percentage of primary follicles and almost all of the growing follicles 3 to 7 days after transplantation and before development of angiogenesis [7].

Steroidogenesis, proliferation, and differentiation of follicular granulosa cells of growing preovulatory stages of ovarian follicles are induced by follicle-stimulating hormone (FSH). However, primordial follicles' initial development is FSH independent [8], but FSH acts as survival factor in serum-free ovarian cortical tissue culture and during primordial follicular transition to primary and secondary follicles [9]. In addition, coordination of germ line and somatic compartments of follicle development in mouse is done by FSH [10]. FSH action in adult mouse can be induced using pregnant mare serum gonadotropin (PMSG), a chorionic gonadotropin hormone of pregnant mare. PMSG superovulation can serve as a good model to understand the probable mechanism of FSH action in follicular development [11].

With that in mind that harvesting of mature oocytes for in vitro fertilization process increases the chances of reproductive success, PMSG is currently used for production of mature superovulated oocytes for in vitro fertilization of valuable species including endangered ones. In addition, it is not known if the transplanted preserved ovaries can respond to the superovulation to achieve this goal of harvesting higher number of matured oocytes. The aim of the present study was to (1) assess superovulation with PMSG on transplanted ovary as an indicator of posttransplantation normal activity of antral follicles, (2) evaluate histomorphometrically the effect of posttransplantation ischemia on different follicular stages, and (3) evaluate the effect of recovery time on follicular growth after autologous transplantation of murine ovaries by the induction of superovulation using PMSG.

## 2. Materials and Methods

### 2.1. Animals

The experimental study was approved by Ethics Committee of School of Veterinary Medicine, Shiraz University. Thirty-six female adult Balb/c mice weighing approximately 25–30 g were provided from Laboratory Animal Center, Shiraz University of Medical Sciences. The animals were kept at 23 ± 1°C and 55 ± 5% relative humidity with 12 h light/dark cycle. They were given standard pellet and water* ad libitum* during experimental period.

The mice were randomly divided into 6 equal groups (*n* = 6), four transplantation groups and two control groups (Table 1). The transplantation groups included two transplantation (14 and 21 d) groups and two PMSG/transplantation (14 and 21 d) groups. The control groups were subdivided to a positive control PMSG group and a negative control group. The mice were entered into study on day of diestrus using vaginal smears. In PMSG positive control group, the mice received single intraperitoneal injection of PMSG (5 IU, Pregnecol, Bioniche Animal Health (A/Asia) Pty Ltd., Armidale, NSW, Australia) and 48 h later the animals were euthanized with ether and cervical dislocation. In the transplantation groups (14 and 21 d), the ovarian autotransplantation was done on both sides of spinal cord during the diestrus phase. After 14 d and 21 d, the mice of transplantation groups were sacrificed. In the PMSG/transplantation groups (14 and 21 d), the same autotransplantation procedure was performed and after 12 d in the first group and 19 d in the second one PMSG (5 IU) was intraperitoneally injected and 48 h later the mice were euthanized. In the negative control group, surgery was not performed and the mice were sacrificed in the estrus phase. Stages of estrus cycle were determined based on vaginal smear method [12].

### 2.2. Ovarian Autotransplantation Surgical Method

Surgical procedures were performed under sterile conditions and in a 24°C temperature operating room. The diestrus mice were weighed and anesthetized with an IP injection of ketamine (100 mg/kg, Alfasan, Woerden, Netherland) and xylazine (10 mg/kg, Alfasan, Woerden, Netherland). Surgical area of abdomen and lateral lumbar region of the mice were surgically prepared. Both ovaries of the mice were removed from a midline abdominal incision and transferred to a sterile dish filled with prewarmed (39°C) sterile saline. Adipose and connective tissues were carefully removed from ovary using a stereomicroscope (SZM, Optika, Italy). The abdominal muscles and skin were sutured with a standard two-layer closure using a simple continuous suture pattern. Then, paralumbar incisions were made on both sides, parallel to the lumbar spinal cord. The ovaries were then grafted into the dorsal lumbar muscles and skin was routinely closed. Oxytetracycline spray was applied on the incision site. Animals were placed in individual controlled 25°C temperature recovery cages.

### 2.3. Histological Evaluation of Ovaries

On the day of sampling, animals were euthanized with ether and ovaries of control and PMSG groups and the transplanted ovarian tissues with their surrounding muscles of transplanting groups were removed. The tissues were fixed in fresh 10% buffered formalin solution in room temperature. After that they were implanted in paraffin. Ethanol and xylene were used for dehydration step. Samples were embedded in paraffin wax and serial sections at thicknesses of 20 *μ*m were performed. During the block sectioning, serial sections were checked until the ovarian tissues appeared in the paraffin section. That was selected as the first section of ovary and the 10th section of every 10 consecutive slices were selected until the observation of the last section with ovarian tissue in the paraffin block. Selected sections were deparaffinized at 60°C and dehydrated in graded concentrations of xylene and ethanol rehydrated in room temperature and stained with hematoxylin and eosin stain.

### 2.4. Histomorphometric Analysis

Follicle types in ovarian sections were defined as previously explained [13] and the numbers of primary, secondary, and tertiary follicles were counted on light microscope (CX21, Olympus, Japan). Sections were also microscopically photographed with an adjusted digital camera (AM423U Eyepiece Camera, Dino-Eye, Taiwan) and Dino Capture 2.0 software (AnMo Electronics Corporation, New Taipei City, Taiwan). The area of total ovary, corpora lutea, and total follicles of each section were measured by drawing their scope using Digimizer software (MedCalc Software bvba; Mariakerke, Belgium).

Moreover, the volume of the ovary, developing follicles of all stages, and corpora lutea of all groups (*V*) were calculated according to the elliptical cone volume formula: *V* = *πD* ^2^ *h*/6, where *π* is equivalent to 3.14, *D* indicates the larger diameter, and *h* indicates the smaller diameter of the ovary, follicles, and corpora lutea. The mean follicle volume (*v*) was measured by taking the average of volume of ovarian tertiary follicles of all stages, according to the following formula: *v* = *V*/*N*, where *N* indicates the numbers of ovarian developing follicles of all stages. Furthermore, stromal volume (*V*_*S*_) was calculated according to the following formula: *V*_*S*_ = *V*_*O*_ − *V*_*F*_, where *V*_*O*_ is the volume of the ovary and *V*_*F*_ is the volume of the follicles.

### 2.5. Statistical Analysis

The data of histological indices of ovary were subjected to Kolmogorov-Smirnov test of normality and analyzed by one-way ANOVA and LSD post hoc test (SPSS for Windows, version 22, SPSS Inc., Chicago, Illinois). The *P* value of less than 0.05 was considered to be statistically significant. Group means and their standard error were reported in the text and graphs (GraphPad Prism version 5.01 for Windows, GraphPad software Inc., San Diego, CA, USA).

## 3. Results

**Figure 1:**
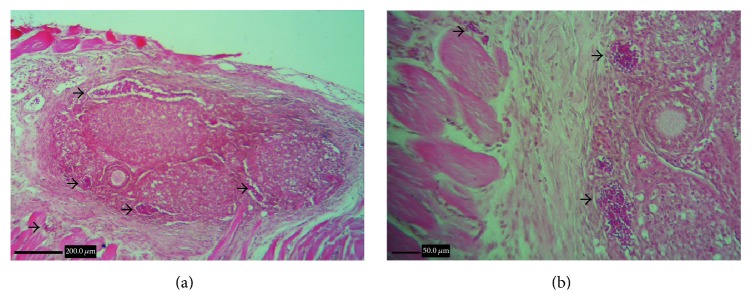
(a) Corpora lutea and secondary follicle in the section confirm ovarian function and folliculogenesis after 21 days' autologous ovarian transplantation in lateral lumbar muscles of mice without superovulation. (b) Angiogenesis, arrows show presence of blood cells in vessels of transplanted ovary and surrounding skeletal muscles. H&E staining.

**Figure 2:**
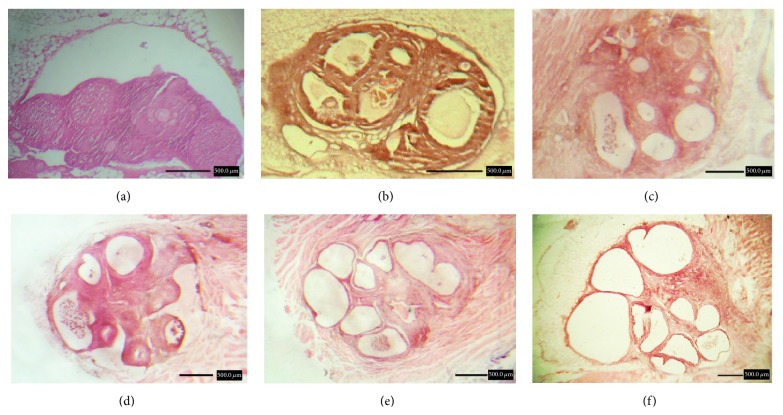
Comparison of the superovulatory effect of pregnant mare serum gonadotropin (PMSG) after autologous ovarian transplantation in mice. Ovaries of groups of (a) control, (b) PMSG, (c) transplantation (14 d), (d) transplantation (21 d), (e) PMSG/transplantation (14 d), and (f) PMSG/transplantation (21 d). H&E staining.

Histological evaluation showed angiogenesis and folliculogenesis after grafting in ovaries in the transplantation and PMSG/transplantation groups (Figure 1). Moreover, in microscopic evaluation of ovaries in the PMSG group and the PMSG/transplantation (14 and 21 d) groups numerous large tertiary follicles were observed, but in the transplantation (14 and 21 d) groups and the control group the number and the size of tertiary follicles were smaller (Figure 2).

**Figure 3:**
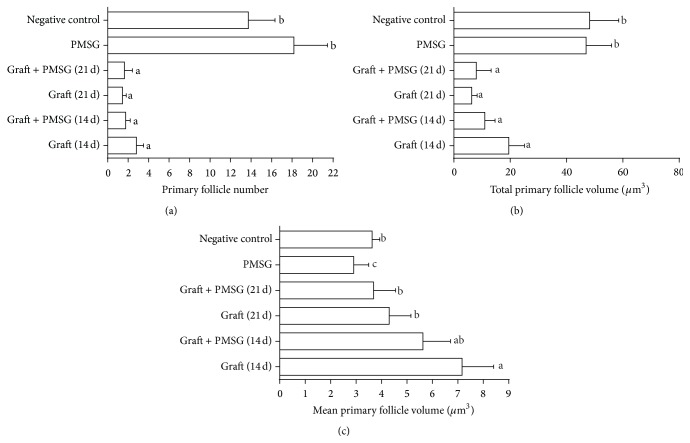
Mean and standard error of histomorphometric analysis of primary follicles (a) total number, (b) total volume, and (c) mean follicle volume in control group, pregnant mare serum gonadotropin (PMSG) group, transplantation (14 and 21 d), and PMSG/transplantation (14 and 21 d) groups after autologous ovarian transplantation and superovulatory effect of PMSG. ^a, b, c^Different superscript letters show significant difference between different groups (*P* < 0.05).

Histomorphometric analysis showed that there was a significant reduction in the number and total volume of primary follicles in the transplantation (14 and 21 d) and PMSG/transplantation (14 and 21 d) groups compared with the control and PMSG groups (*P* < 0.05, Figures 3(a) and 3(b)). The mean primary follicle volume in the transplantation (14 d) group was more than that in the other groups except for the PMSG/transplantation (14 d) group (*P* < 0.05, Figure 3(c)). Also there was a significant decrease in the mean primary follicle volume in the PMSG group in comparison with the other groups (*P* < 0.05).

**Figure 4:**
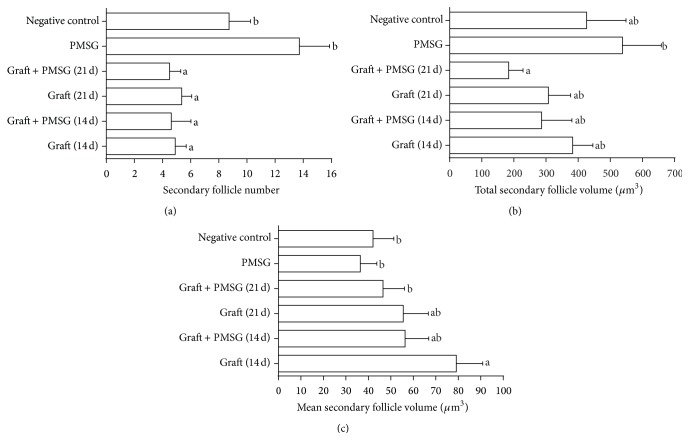
Mean and standard error of histomorphometric analysis of secondary follicles (a) total number, (b) total volume, and (c) mean follicle volume in control group, pregnant mare serum gonadotropin (PMSG) group, transplantation (14 and 21 d), and PMSG/transplantation (14 and 21 d) groups after autologous ovarian transplantation and superovulatory effect of PMSG. ^a, b^Different superscript letters show significant difference between different groups (*P* < 0.05).

Same as primary follicles, there was a significant reduction in the number of secondary follicles in the transplantation (14 and 21 d) and PMSG/transplantation (14 and 21 d) groups compared with the control and PMSG groups (*P* < 0.05, Figure 4(a)). Moreover, the total volume of secondary follicles in the PMSG group was more than the PMSG/transplantation (21 d) group (*P* < 0.05, Figure 4(b)). The mean secondary follicle volume in the transplantation (14 d) group was significantly more than the control, PMSG, and PMSG/transplantation (21 d) groups (*P* < 0.05, Figure 4(c)).

**Figure 5:**
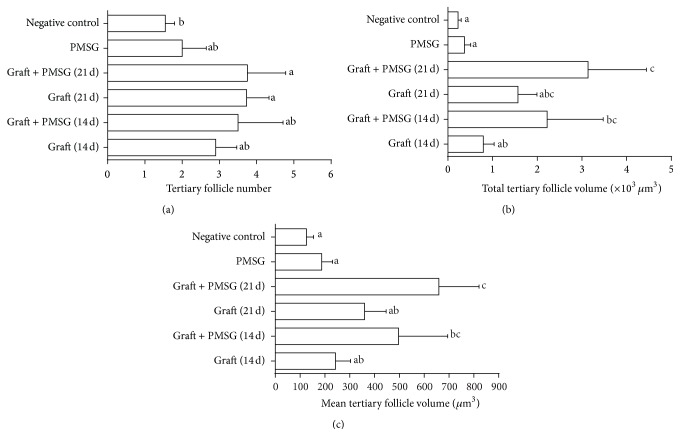
Mean and standard error of histomorphometric analysis of tertiary follicles (a) total number, (b) total volume, and (c) mean follicle volume in control group, pregnant mare serum gonadotropin (PMSG) group, transplantation (14 and 21 d), and PMSG/transplantation (14 and 21 d) groups after autologous ovarian transplantation and superovulatory effect of PMSG. ^a, b, c^Different superscript letters show significant difference between different groups (*P* < 0.05).

The number of tertiary follicles in the transplantation (21 d) and PMSG/transplantation (21 d) groups was significantly more than the control group (*P* < 0.05, Figure 5(a)). The total volume of tertiary follicles in the PMSG/transplantation (21 d) group was significantly greater than the control, PMSG, and transplantation (14 d) groups (*P* < 0.05, Figure 5(b)). The mean tertiary follicle volume in the PMSG/transplantation (14 and 21 d) group was more than the control and PMSG groups (*P* < 0.05, Figure 5(c)).

**Figure 6:**
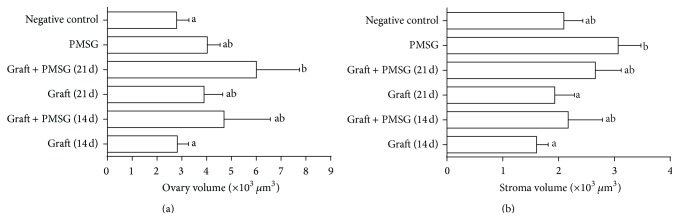
Mean and standard error of histomorphometric analysis of (a) ovarian volume and (b) stroma volume in control group, pregnant mare serum gonadotropin (PMSG) group, transplantation (14 and 21 d), and PMSG/transplantation (14 and 21 d) groups after autologous ovarian transplantation and superovulatory effect of PMSG. ^a, b^Different superscript letters show significant difference between different groups (*P* < 0.05).

Ovary volume in the PMSG/transplantation (21 d) group was more than the control and transplantation (14 d) groups (*P* < 0.05, Figure 6(a)). Ovarian stromal volume in the PMSG group was more than the transplantation (14 and 21 d) groups (*P* < 0.05, Figure 6(b)).

## 4. Discussion

In the present study, the impact of transplantation ischemia on survival and development of different follicular stages following whole ovary heterotopic autotransplantation were histomorphometrically evaluated after 14 and 21 d. The results indicated that survival and development of different follicular types were influenced by ischemia. Reduction in the number of primary and secondary follicles in the transplantation and PMSG/transplantation groups after 14 and 21 d showed the effect of ischemia on these follicular stages. However, the number and volume of these follicles decreased after grafting but estimated individual size of both types was increased after 14 days and again was decreased on day 21. Simultaneously, during the same period, increase of the number and volume of tertiary follicles showed follicular growth continued and was enhanced after heterotopic transplantation. Consistent with our findings, Xie et al. [3] recently showed that healthy rate of follicles and the number of follicles with positive proliferating cell nuclear antigen in primary follicles decreased 1 month after orthotopic autografting of the rabbit ovaries. Early follicular development is regulated by ovarian autocrine/paracrine regulators and interactions between oocyte-granulosa cells, ovarian stromal cells, and theca cells affect this process [14]. Grafting could induce deactivation of primordial follicles [15]. Therefore, decrease in number of primary follicles can be affected by cessation of primordial follicle growth. Our findings indirectly and directly may indicate that follicular growth and development in early stage (primordial and primary follicles) were more influenced by ischemia in comparison with late stages (secondary and tertiary follicles), and follicle reservoirs in primordial and primary stages cannot be well replaced after transplantation ischemia.

In this study, we observed that ovarian tissue survived and follicles grew in muscular spaces of back muscle. A rapid blood supply can prevent loss of follicular pool and cessation of folliculogenesis after ovarian transplantation may reduce the follicular quality and response to hormonal alterations. Therefore, in this study the effect of intraperitoneal injection of PMSG on follicular growth after ovarian transplantation was evaluated as an index of presence of ovarian blood supply, angiogenesis, and folliculogenesis especially after primordial follicular stage. Significant differences in the primary, secondary, and tertiary follicle number and volume after transplantation and superovulation, which indicated the time despite the positive role in follicular survival and better angiogenesis, have a significant impact on follicular growth and maturation in response to superovulation. Anatomically, primary follicles in rodents are small and located very close to the surface of ovary [16]. On the other hand, oocyte metabolism was higher in primary follicles than at any subsequent stage [17]. Therefore, more reduction of primary follicles than secondary ones after one week (time between two samplings) may be the result of the effect of posttransplantation ischemia on reduction of early stages of follicles.

Complete removal of ovarian fat tissue before transplantation enhanced revascularization via facilitation of cell infiltration from the high blood supply muscular tissue. Formation of new blood vessels was initiated by elongation, sprouting, intussusception, or the incorporation of circulating endothelial cells of preexisting vasculature [18]. Most of these processes can be involved in angiogenesis of ovary [19]. Ischemic damage of ovarian tissue is unavoidable during postgrafting period and its effect is reduced after neo- and revascularization. Vascular connections between the murine ovary and transplanted site were observed 5 days after transplantation [20].

A cohort of primordial follicles within 10 to 12 d reaches the secondary follicle stage and by 6 to 12 d develop to the large antral stage in mice [21]. Considering the 5 d of posttransplantation angiogenesis, in the first sampling of 14 d, the evaluated tertiary follicles were the developed follicles from the primary and secondary follicles which suffered from posttransplantation ischemia, while in the second sampling of 21 d the sectioned tertiary follicles were related to the developed follicles from the primary and secondary follicles after angiogenesis. Therefore, the increase in mean of number or volume of tertiary follicles after 21 d in comparison with 14 d sampling in transplantation groups can be explained.

## 5. Conclusions

Primary and secondary follicles can survive after autologous transplantation but their reservoirs gradually get diminished by increasing the time of transplantation. However, number of tertiary follicles and their response to superovulation increased over time after transplantation. Therefore, it seems that early collection or superovulation of transplanted ovaries may result in more tertiary follicles.

## Figures and Tables

**Table 1:** Groups and procedures for evaluation of superovulation effect on follicular development after autologous ovarian transplantation in mice.

Groups	Transplantation	PMSG injection and time	Day of sampling
Negative control	−	−	In the estrus phase
PMSG	−	+ (In the diestrus phase)	2 d after injection
Graft (14 d)	+	−	14 d after transplantation
Graft (21 d )	+	−	21 d after transplantation
Graft + PMSG (14 d)	+	+ (12 d after transplantation)	14 d after transplantation
Graft + PMSG (21 d)	+	+ (19 d after transplantation)	21 d after transplantation
